# Evolution and ecology of body size in the world’s largest bats

**DOI:** 10.1098/rspb.2025.0743

**Published:** 2025-07-16

**Authors:** Francisca C. Almeida, Kristofer M. Helgen, Nancy B. Simmons, Norberto P. Giannini

**Affiliations:** ^1^ Department of Ecología, Genética y Evolución, Universidad de Buenos Aires, Buenos Aires, Capital Federal, Argentina; ^2^ IEGEBA, CONICET, Buenos Aires, Argentina; ^3^ Australian Museum, Sydney, Australia; ^4^ Bernice Pauahi Bishop Museum, Honolulu, Hawai'i, USA; ^5^ American Museum of Natural History, New York, NY, USA; ^6^ CONICET-Fundacion Miguel Lillo Unidad Ejecutora Lillo, San Miguel de Tucumán, Tucumán Province, Argentina; ^7^ Universidad Nacional de Tucuman Facultad de Ciencias Exactas y Tecnologia, San Miguel de Tucumán, Tucumán Province, Argentina

**Keywords:** flying fox, phenotypic evolution, phylogenetic comparative methods, islands, Pteropodinae, *Pteropus*, Orstein–Uhlenbeck model

## Abstract

*Pteropus* and closely related flying fox genera in the subfamily Pteropodinae represent a remarkable radiation of insular taxa. Comprising more than 80 species, the group includes the largest living or extinct bat species. Exceptional vagility has allowed these bats to colonize numerous Pacific and Indian Ocean islands, where they play crucial ecological roles in maintaining ecosystems. It has long been noted that on islands where multiple species coexist, there is a tendency for size differentiation among them. We investigated this pattern in depth using skull length as a proxy for body size and analysing hundreds of data points across most species and islands. We employed a phylogenetic framework to evaluate the evolutionary processes driving size variation in *Pteropus* and Pteropodinae. We updated the molecular phylogeny to include most pteropodine species and applied phylogenetic comparative methods to evaluate different models of phenotypic evolution. Results suggest that natural selection, most likely through character displacement in islands, played a significant role in the evolution of body size in Pteropodinae. Additionally, other processes such as species sorting and ecological release may also have contributed to the observed pattern of size evolution.

## 1. Background

The bat subfamily Pteropodinae comprises 85 species of flying foxes classified in nine genera, most of which inhabit islands of the Pacific and Indian oceans, with a few species distributed in continental areas of South Asia and Australia [1,2]. The majority of pteropodine species feed on fruits and floral parts, with the exception of species of the genera *Melonycteris* and *Nesonycteris*, which are specialized nectar feeders [3–5]. As a consequence, pteropodines play important ecological roles in their habitats as pollinators and seed dispersers wherever they occur [6,7]. Probably because of compounding vulnerabilities relating to island habitats, many species are endangered, chiefly due to hunting, habitat loss and human–wildlife conflict over local resources; and six species, all island endemics, have become extinct in historical times [8–11].

The most speciose pteropodine taxon is the iconic genus *Pteropus*, representing an explosive radiation of at least 64 species of flying foxes [1,2,12,13]. The other pteropodine genera (*Acerodon*, *Neopteryx*, *Styloctenium*, *Pteralopex*, *Mirimiri*, *Desmalopex*, *Melonycteris* and *Nesonycteris*) are much less diverse, with a maximum of five recognized species each [2]. Molecular dating analyses have timed the origin of *Pteropus* to around 7−8 Ma, with some speciose groups (e.g. the *hypomelanus* group) being only about 2 million years old [14–16]. A study of diversification rates in mammals found that *Pteropus* had the second highest rate of increase among all mammal genera that diversified in the last 10 million years [12]. Those dating estimates suggest that the current distribution of *Pteropus* species was achieved primarily by dispersal over the past 2 Myr across the majority of islands of Wallacea, the Philippines, the south-west Pacific and the Indian Ocean, which were already separated by significant expanses of water [17]. Phylogenetic trees indicate, with a few exceptions, that species co-occurring in the same islands are not sister species, pointing to allopatric speciation following dispersal and colonization as the main diversification mechanism of the group [16,18].

*Pteropus* and its sister genus, *Acerodon*, include the largest known bat species, with adult individuals of the largest species (e.g. *Acerodon jubatus*, *Pteropus vampyrus*) weighing up to 1500 g and reaching up to 1.8 m in wingspan [19]. On the other extreme, the smallest *Pteropus* species, *Pteropus molossinus*, has an average adult body mass of just over 100 g [20]. Studies on body size evolution in Chiroptera suggest that this character is highly constrained in most bat lineages, probably due to physiological requirements imposed by echolocation [21–23]. The release of these constraints in Pteropodidae with the loss of laryngeal echolocation [24] resulted in highly dynamic changes in body sizes during evolution within this family; this pattern is evident in Pteropodinae and particularly in *Pteropus*, for which phylogenetic comparative methods have detected several optimum shifts in body size throughout its evolutionary history [23].

The high taxonomic and morphological diversity of pteropodines may be associated with the predominantly insular distribution of its component species. Restricted island areas result in small populations, which are subject to founder effects, genetic drift, accelerated evolutionary rates and selection regimes that differ from continental settings [25,26]. Limited resources may lead to competition, resource shift and adaptation, which combined with alternating circumstances of sympatry and allopatry, may promote adaptive radiation in archipelagos [27]. Moreover, insular environments are often the setting for evolutionary changes in body size in response to selective forces imposed by particular ecological characteristics that may include, besides limited resources and challenges to dispersal, a reduced number of predators and/or interspecific competitors [28,29]. Island environments are very heterogeneous, however, and their ecology is influenced both by their areas and their distances to other islands and continents (degree of isolation) [30].

In his landmark contribution, the monograph *Catalogue of the Chiroptera*, Andersen [31, p. 97] observed that sympatric *Pteropus* species frequently diverge conspicuously in body size, in such a way that ‘The length of the forearm of a specimen to be determined, together with the precise locality given on its label, will therefore in most cases help the non-specialist to an easier and quicker identification than the necessarily long and complicated dichotomic key to the species*’*. This pattern may be explained chiefly by two evolutionary processes: species sorting and character displacement [32,33]. In the former case, also known as biased extinction, size differences evolve prior to island colonization and sympatry results in the selective exclusion of one of the similar-sized species, leaving only those that do not overlap in size. In the case of character displacement, species with similar sizes could have colonized the same island, but one or both evolved towards an increase or reduction in size that would minimize overlapping, avoiding competition for the same limited resources and leading to co-existence. In this case, size differences would have evolved after colonization.

Here, we present an analysis of body size variation in *Pteropus* and related frugivorous pteropodine species to test hypotheses about the drivers of size evolution in this clade. With this goal, we updated the molecular phylogeny of pteropodines, increasing the number of species using available molecular data from several mitochondrial and nuclear loci. We reviewed Andersen’s [31] observations using an extensive dataset of cranial measurements and up-to-date data on species distributions. Using this dataset and the updated phylogeny, we applied phylogenetic comparative methods to estimate ancestral sizes and phenotypic disparity through time. Finally, we assessed the role of various evolutionary forces in the evolution of body size in *Pteropus* and the family Pteropodinae by comparing the fit of different models of character evolution to the data, and evaluating patterns of intraspecific size variation.

## 2. Methods

### (a) Data

We obtained and curated three types of data from pteropodine species: DNA sequences, geographical distributions and skull condylobasal length (CBL). DNA sequences obtained in previous studies (mainly [16,18]) were retrieved from the GenBank, representing five loci: cytochrome b (*CYTB*), mitochondrial control region (D-loop), recombination activation gene 1 (*RAG1*), recombination activation gene 2 (*RAG2*) and an intron of the signal transducer and activator of transcription 5A gene (*STAT5A*). At least one of these loci was obtained for each taxon included in our analyses. The final taxonomic sample for the phylogenetic analysis comprised 53 *Pteropus* species (93% of the currently recognized diversity), multiple subspecies from three species (*Pteropus pelewensis*, *Pteropus tonganus* and *Pteropus alecto*), 14 species belonging to other pteropodine genera and four non-pteropodine species as outgroups (electronic supplementary material, table S1). The geographical distributions of each species were mostly obtained from the most recent review of Pteropodidae [34], although occasionally additional literature (chiefly [35]) was consulted (electronic supplementary material, table S2).

We used CBL (the distance from the posterior edges of the occipital condyles to the anterior edge of the premaxilla) as a proxy for overall body size. This measurement was chosen because it is less variable than other measures (e.g. mass, which varies with conditions such as pregnancy or time since last meal), is available for more specimens and species and can be considered a good proxy of size when only skull data are available (see below). Skull measurements were mostly obtained from museum specimens, although for some species we relied on measurements obtained from the literature (electronic supplementary material, table S3). We gathered CBL data for adult specimens of 61 *Pteropus* nominal taxa plus 14 non-*Pteropus* pteropodine species, and pooled data from males and females, as pteropodines generally exhibit negligible sexual size dimorphism (although this is present but subtle in a few species, e.g. [36,37]). In total, the number of CBL measured per species varied from 1 (*Pteropus niger*) to 123 (*Acerodon jubatus*). Body length (35 species) and mass (49 species) data were obtained from PanTHERIA [38] to assess the actual degree of correlation of CBL with other measures of body size. To check for differences in body sizes both between species and between different populations of one species, we used ANOVA and the post-hoc Tukey’s HSD test. All statistical analyses were performed with R [39].

### (b) Phylogeny and dating

Sequences from each locus were separately aligned with MAFFT [40], and the resulting alignments were concatenated with Mesquite [41]. We employed the program IQ-TREE [42] to determine the best partitioning scheme, taking into account the different codon positions for the three coding loci, and to run maximum likelihood tree searches with 1000 bootstrap resampling to obtain clade support. Dated trees were obtained with the package BEAST [43], using previously estimated node age ranges for calibration [44]. We applied an uncorrelated lognormal clock with a Yule speciation process and set a prior age for *Pteropus* using a normal distribution with a mean of 8.7 mya and a standard deviation of 0.8 (95% confidence interval between 7.1 and 10.2 mya). The analysis was run, checked for convergence and summarized as described in Amador *et al.* [44].

### (c) Phylogenetic comparative methods

As a first approach to understanding the evolution of size in pteropodines, we tested whether average CBL had significant phylogenetic signal based on both Pagel’s λ [45] and Blomberg’s *K* [46], using the R package *phytools* [47]. We also estimated ancestral CBL for each internal node using residual maximum likelihood (REML) with the R package *ape* [48]. We then assessed the fit of different models of continuous character evolution to the CBL data using the dated pteropodine tree and average CBL per taxon. We examined the Brownian motion (BM), early burst (EB) and Ornstein–Uhlenbeck (OU) models using the function *fitContinuous* of the package *geiger* [49–52]. Model fits were contrasted using AIC weights [53].

The application of the OU model in a phylogenetic context, having as hypotheses different arrangements of optima along the branches of a tree, is known as the Hansen model [54]. We applied the Hansen model, as implemented in the R package *OUwie* [55], to address the role of selection in the evolution of body size in Pteropodinae [56]. To fit a multi-optimum (Hansen) model, each species in the dataset was assigned to a category that may have a size optimum different from the optimum of other categories. As we wanted to assess the role of character displacement, we implemented categories that were based on sympatry status and relative size among sympatrically occurring species (electronic supplementary material, table S2). Each area (continental area, island or archipelago) inhabited by pteropodines is home to up to five sympatrically occurring species; thus, we opted for a basic scheme with three optima, as follows. In the simplest scenario, when three species were found in sympatry on an island, those were classified into either the ‘small’ (S), ‘medium’ (M) and ‘large’ (L) categories, correspondingly. More complex scenarios arose when the number of species was different from three. When only two species were present on an island, we took into account their size ranges and the size difference between the two species. Thus, those islands had a combination of ‘S’ and ‘M’, ‘M’ and ‘L’ or ‘S’ and ‘L’ species. When four or more species were sympatric, species were sorted into size categories, with at least one size category containing more than one species. Widespread species found in many archipelagos and occurring in sympatry with many different species in different areas (e.g. *P. hypomelanus* and *P. vampyrus*) were assigned to the category into which they fit most commonly, taking into account all the islands/archipelagos where they occur.

For the few species that are not sympatric with any other *Pteropus* or other pteropodine taxa, we tested two alternative scenarios: (i) isolated species included in the ‘M’ category (i.e. the size category with mean CBL that was closer to the estimated ancestral CBLs; see Results) or (ii) isolated species were placed in a separate category, ‘isolated’ (‘I’) not dependent on size. Both extant and recently extinct species (e.g. *P. subniger* and *P. pilosus*) that were not represented in the phylogeny due to a lack of molecular data were taken into account when placing extant species into size categories included in the model fitting analysis.

We compared the fit of five main evolutionary models: BM, BMS (BM assuming different variation parameter for the different size categories), OU1 (OU with one optimum) and OU3 with multiple optima according to the three categories scheme (‘L’, ‘M’ and ‘S’) and OU4 according to the four categories scheme (‘L’, ‘M’, ‘S’ and ‘I’). For each of those schemes, we also fitted alternative OU models, where the parameters alpha (pull towards the optimum) and/or sigma (variance) varied among categories. As an additional test, we applied a scheme similar to scheme 3, in which species were randomly assigned to the categories ‘L’, ‘M’ and ‘S’ (OU3r). We then compared the fit of the different schemes applying Akaike’s information criterion for finite samples (AICc) [53].

The Butler and King [54] method, as implemented in *OUwie,* requires that the internal nodes be assigned to one of the categories. To avoid bias, we ran the model fit for each scheme several times, considering different categories (‘S’, ‘M’, or ‘L’) for the internal nodes. Model fit was conducted with both the mean measurements for each taxon and their logarithmic transformations. All comparative analyses were done for two datasets: (i) *Pteropus* species only, and (ii) frugivorous pteropodines (Pteropodinae excluding the nectarivorous taxa *Melonycteris* and *Nesonycteris*). The latter analysis was conducted in this way, given that all frugivorous pteropodines are expected to use, and probably compete for, fruit resources when in sympatry on islands.

## 3. Results

### (a) Phylogeny

The topologies of the phylogenetic trees obtained with IQtree and BEAST (dated) were very similar (electronic supplementary material, material, figures S1 and S2). All genera were recovered with very high support (>98% bootstrap support), although some intergeneric relationships had below 95% bootstrap values. Within the genus *Pteropus*, several relationships received high statistical support, including previously proposed species groups (see table 1 and §4). Some relatively recent interspecific relationships (i.e. those with node ages <2 million years) varied in position between trees and/or had low bootstrap values, suggesting insufficient information in the matrix to resolve these recent cladogenetic events.

**Table 1.** Results of evolutionary model fit for *Pteropus*. BM, BMS, OU1, OU3 and OU4 were the different models compared (see text for descriptions). Shown for each model is: logLik = log likelihood, AICc = Akaike information criterion, 𝜎 = intensity of stochastic evolution, 𝛼 = rate of evolution towards the optima (𝜃), 𝜃 = estimated optimum for each category with its standard error and mean and standard deviation of CBLs in each category.

	BM	BMS	OU1	OU3	OU4
log_L	−203.03	−202.49	−198.93	−190.96	−181.13
AICc	410.28	413.75	404.33	393.13	375.97
σ	33.9	38.5, 33.5, 0	56.5	100.0	52.0
α			0.27	1.14	0.60
θ1 (s.e.)	53.6 (7.1)	52.7 (6.7)	55.8 (2.7)		
θL (s.e.)				66.6 (1.8)	93.4 (5.6)
θM (s.e.)				51.9 (2.6)	65.1 (4.7)
θS (s.e.)				44.9 (2.4)	47.5 (3.2)
θI (s.e.)					54.4 (2.0)
L ± s.d.				70.6+5.6	71.0+5.6
M ± s.d.				59.0+7.5	59.3+4.5
S ± s.d.				49.3+5.5	49.3+5.5
I ± s.d.					57.2+9.3

### (b) Condylobasal length variation

**Figure 1.**
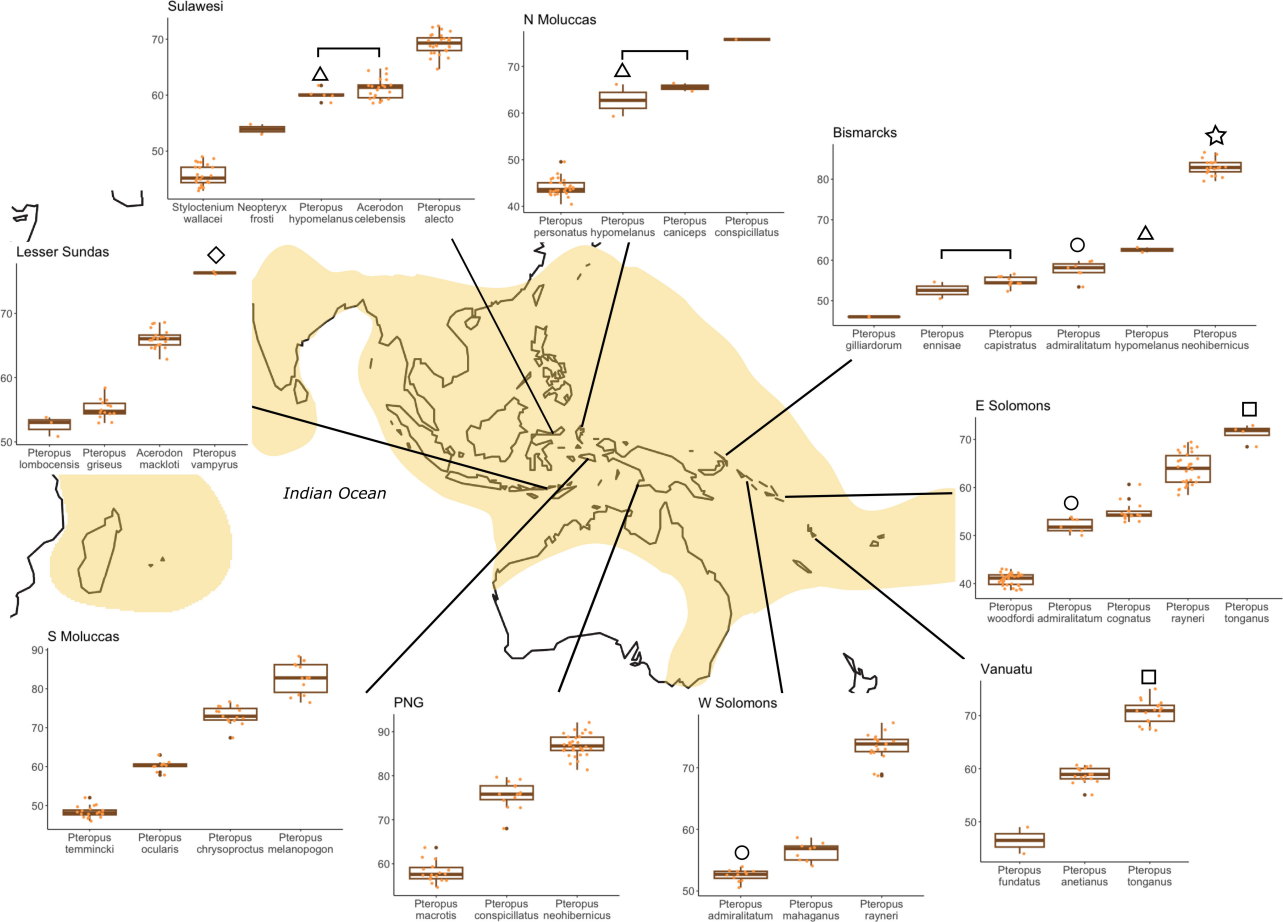
Box plots showing CBL statistics of species co-inhabiting the same archipelago (although not all species are strictly sympatric, since some inhabit different islands: e.g. *P. capistratus* and *P. ennisae*; or *P. lombocensis* and *P. griseus*). Symbols indicate widespread species. Brackets show species size comparisons that were not significantly different (Tukey’s HSD test).

Average CBL in frugivorous pteropodine species varied from 38.8 mm in *P. molossinus* to 84.0 mm in *P. neohibernicus*. CBL was significantly and positively correlated with both body length (*R* = 0.88) and mass (*R* = 0.93), proving to be a good proxy of body size (electronic supplementary material, figure S3). We examined the CBL and geographical distribution data and confirmed the pattern first observed by Andersen [31], finding little overlap in size between sympatric *Pteropus* species (figure 1; electronic supplementary material, figure S4). For these comparisons, we took into account only the measurements of specimens from each specific island/archipelago, which meant that in some cases, measurements of only a few specimens were available, thus reducing the power of the tests. Among sympatric species, the only case of significant size overlap involving *Pteropus* species that we detected was between *P. hypomelanus* and *P. caniceps* on Halmahera and its satellites (Moluccas).

### (c) Evolution of condylobasal length in *Pteropus*

**Figure 2.**
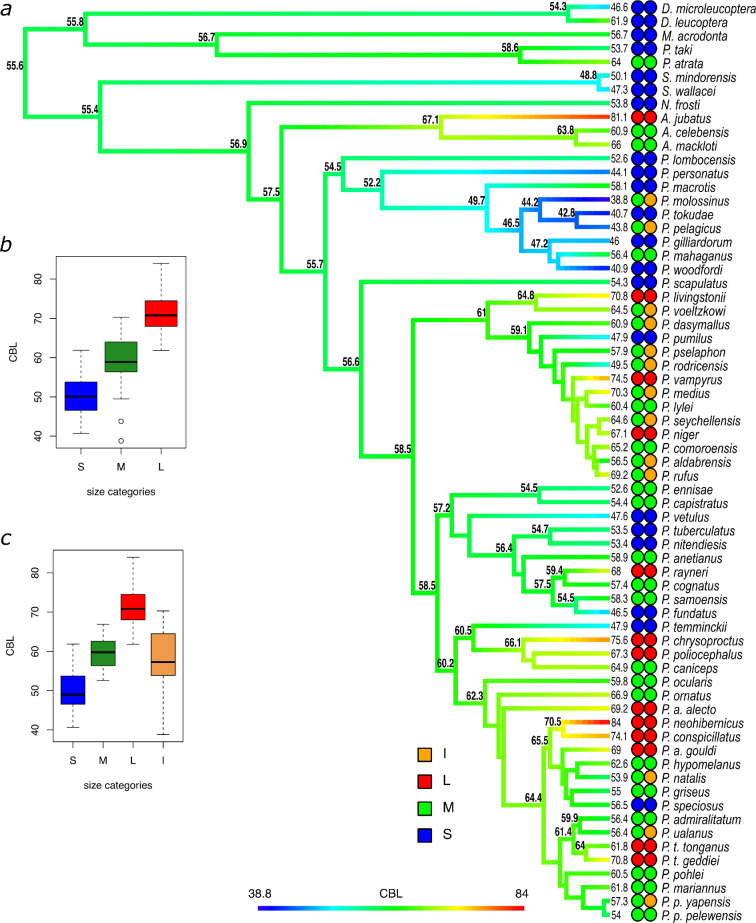
(*a*) Phylogenetic tree showing the evolution of CBL in frugivorous pteropodines with estimated ancestral CBL for internal nodes, mean CBL per terminal taxon and category for the model fitting analysis (colored circles). (*b*) Boxplot showing the distribution of CBL of frugivorous pteropodines in each category of the model fitting analysis, corresponding to the three categories scheme (first column of circles next to the tree terminals). (c) The same as in b but corresponding to the four categories scheme (second column of circles next to the tree terminals).

**Figure 3.**
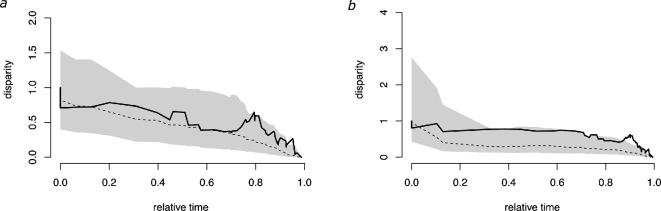
(*a*) Disparity through time plot (black line) for the genus *Pteropus* with null range simulated under Brownian motion (grey area). (*b*) As for (*a*) but for all frugivorous pteropodines represented on the tree.

The estimated ancestral CBL of *Pteropus* was 55.6 mm. Size shifts, including both increases and decreases, were detected throughout the phylogeny, and in all major clades including internal and external branches (figure 2*a*; electronic supplementary material, figure S5). Accordingly, a relatively constant rate of CBL disparity was recovered across time, although the disparity tended to be closer to the highest values of the null distribution range in the last 2.5 million years of *Pteropus* evolution (figure 3*a*). This pattern is more obvious when non-*Pteropus* species are included in the analysis (figure 3*b*). The morphological disparity index was 0.10 (*p* = 0.71) and 0.33 (*p* = 0.953) for *Pteropus* and frugivorous pteropodines, respectively. Pagel’s delta was 3 for both datasets and significant, reflecting important morphological disparity within clades. Nevertheless, two tests revealed a relatively weak, albeit significant phylogenetic signal in CBL in the genus *Pteropus* (λ = 0.66, *K* = 0.54, *p* = 0.0002). The OU model had the best fit to the data as compared with the BM and EB models, with an AIC weight of 0.93.

### (d) Model fitting in *Pteropus*

The species categorization based on sympatry/allopatry and CBL for the different schemes are shown in figure 2 (electronic supplementary material, table S2). The CBLs of ‘small’ *Pteropus* species varied from 40.9 mm in *P. woodfordi* to 58.1 mm in *P. macrotis*, with an average of 49.3 cm. ‘Medium’ species exhibited CBL varying from 52.6 mm (*P. ennisae*) to 66.9 mm (*P. ornatus*), overlapping with the ranges of sizes of both ‘small’ and ‘large’ species across the phylogeny. The average ‘medium’ size was approximately 59 mm and the optimum (according to the OU model) varied, depending on the scheme used, from 52.4 to 61.3 mm. Allopatric (‘I’) species had similar mean CBL as compared to sympatric ‘M’ species, although variation was significantly larger in the former category, which included the smallest species, *Pteropus molossinus* (38.8 mm) but also *P. medius* (70.3 mm), a relatively large species from the Indian subcontinent. The *Pteropus* species placed in the ‘large’ category had an average CBL varying from 61.8 mm (*P. tonganus tonganus*) to 82.9 mm (*P. melanopogon*, not included in model fitting analysis). In the past, *P. tonganus tonganus*, the smallest among the ‘large’ species, was sympatric with the larger *Pteropus coxi*, an extinct species from Samoa that was not included in our analysis because the single available skull is broken and CBL cannot be measured, and it lacks DNA sequences. Therefore, in Samoa, *P. tonganus tonganus* occupied the relative position of a ‘medium’ species, although in other islands of its distribution, it is the largest species recorded.

The results of model fitting analysis shown in table 1 were based on analyses where the internal nodes were assigned to the category ‘M’, as model fits were not affected by the category applied to the internal nodes (results not shown). Those results were obtained using actual CBL measurements, although similar results were obtained with log-transformed data (not shown). The best fitting scheme was scheme 4, which comprised four categories, ‘small’, ‘medium’, ‘large’ and ‘isolated’, rejecting a purely neutral process and pointing to the influence of sympatry and selection in the evolution of size in *Pteropus*. For the OU4 model, however, the analysis with *OUwie* resulted in a warning that the amount of data could be low for the number of parameters to be estimated, although the analysis apparently ‘arrived at a reliable solution’ according to the output. The same was true for all models with different alpha and sigma parameters for each category (electronic supplementary material, table S4), although their fits were never better than those of the models considering the same parameter values for all categories. The OU3r scheme, where categories were randomly assigned, had a worse fit than the OU3 (and OU4) in all 1000 simulations (AICc between 399.1.3 and 409.1; electronic supplementary material, figure S6).

### (e) Frugivorous pteropodines

**Figure 4.**
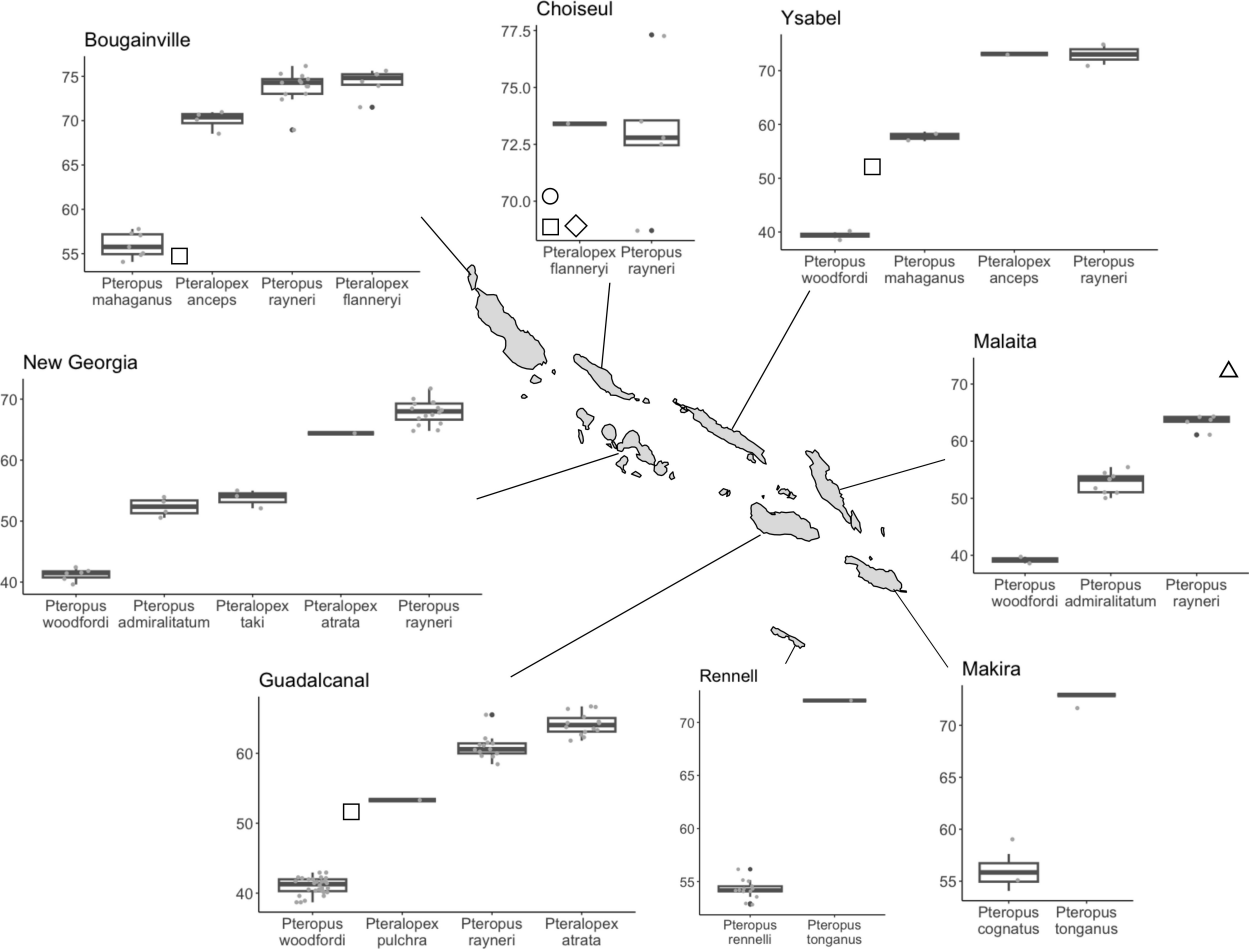
Box plots showing pteropodine species size (CBL) statistics per island/island group of the Solomon archipelago. Symbols represent species for which we did not have CBL measurements: (△) *P. tonganus* in Malaita, (▯) *P. admiralitatum* in Bougainville, Choiseul, Ysabel and Guadalcanal, (◇) *P. mahaganus* and (◯) *Pteralopex anceps* in Choiseul. Asterisks were placed based on CBL recorded in other populations of the same species to indicate where the missing species might be expected to fall relative to congeners on each island/island group.

When we compared the CBL of species of other pteropodine genera in addition to *Pteropus* species, we found the same general pattern of little or no size overlap. Interestingly, some non-*Pteropus* taxa seem to fit in size gaps not filled by *Pteropus* species in some areas. For example, in the Lesser Sundas, *Acerodon mackloti* occupies the medium-sized category; in Sulawesi, *Neopteryx frosti* and *Styloctenium wallacei* are the two smallest species and in the Philippines, *Desmalopex* species occupy the small size ranges (figure 1; electronic supplementary material, figure S4). Exceptions to the general pattern of no size overlapping were observed between some pairs of sympatric species, such as *Acerodon celebensis* and *P. hypomelanus* in Sulawesi (figure 1), *Pteralopex* species with other species of the same genus and *Pteropus* species in the Solomon Islands (figure 4) and *Mirimiri acrodonta* y *P. samoensis* in Fiji (electronic supplementary material, figure S4).

As all frugivorous pteropodines could be involved in niche sharing (sharing the same food source(s)) and character displacement, we reassessed the size evolution models based on sympatry using the complete pteropodine dataset, with the exclusion of the nectarivorous Melonycterini species. Phylogenetic signal remained significant (*L* = 0.68 and *K* = 0.24, *p* = 0.0002) and the OU4 model again had the best fit (electronic supplementary material, table S5).

Considering all frugivorous pteropodines and the best fitting scheme, we evaluated size changes in each category (electronic supplementary material, table S6). We found that species in the ‘large’ category increased, on average, by 9.6% compared to the size of their reconstructed most recent ancestor. Only one ‘large’ taxon, *P. tonganus tonganus*, showed a slight reduction in size in relation to its direct ancestor, but as already mentioned, it was not the largest species in part of its geographical distribution. Seventeen out of the 20 ‘small’ species exhibited reduction in size, although only nine had decreases of more than 5% with respect to the reconstructed ancestor size. Two ‘small’ species, however, underwent substantial size increases: *Desmalopex leucoptera* from Luzon and *Pteropus macrotis* from Papua New Guinea, both of which were sympatric with very large species. The 21 ‘medium’ species showed much less dramatic changes in size since their divergence, with a net increment of 0.3%. ‘Isolated’ species were also more similar to their most recent ancestors, although with a tendency to size decrease, with an average overall reduction of 3%.

### (f) Intraspecific variation

Intraspecific inter-island size differences can help distinguish between the alternative hypothesis of character displacement and species sorting. An analysis of intraspecific variation showed that most species that occur on more than one island show significant size differences among populations on different islands (e.g. *P. admiralitatum*, *P. alecto*, *P. griseus*, *P. neohibernicus*, *P. rayneri*), with some exceptions (e.g. *P. samoensis*) (electronic supplementary material, figure S7). Nevertheless, for some islands, measurements of only a few individuals were available, and for some species only parts of the distribution were included in our analysis due to limited specimen availability, which may have biased the results. Results in these cases should be considered as ‘best guess’ hypotheses in need of further testing. In general, however, intraspecific intra-island variation was quite low, as indicated by low coefficients of variation (electronic supplementary material, table S7).

The Solomon Islands archipelago is home to a large diversity of species that exhibit an interesting pattern of intraspecific and interspecific size variation (figure 4). *Pteropus tonganus* is larger in the eastern Solomon Islands (72.5 **±** 0.6, *n* = 2) (but also in Vanuatu and Nendo*—P. tonganus geddiei*), where it is sympatric with *P. rayneri rayneri* (63.4 ± 1.3, *n* = 5) so that these two species do not overlap in size where they co-occur (figures 1 and 4; electronic supplementary material, figure S7). *Pteropus tonganus* is smaller in size in Samoa (but also in other Polynesian islands and Fiji—*P. tonganus tonganus*) (61.4 ± 1.3, *n* = 15), where *P. rayneri* is not present, but where it used to co-occur with *Pteropus coxi*, as already mentioned [9]. *Pteropus rayneri* also shows significant size differences among island populations, being larger (70.7 ± 3.4, *n* = 42) in the western Solomon Islands (Bougainville, Choiseul, Santa Isabel, New Georgia), where its populations are not sympatric with *P. tonganus*. The smallest population CBL mean for *P. rayneri* is recorded in Guadalcanal, where *P. tonganus* is not present but another large pteropodine, *Pteralopex atrata,* is the largest species (figure 4; electronic supplementary material, figure S7). It is noticeable that outlier individuals of *P. rayneri* are found in both the east and west Solomon Islands, which could be explained by inter-island migration (figure 4). Another similar pattern found among the Solomon Islands species involves *P. admiralitatum*, a widespread species. This species is smaller in the west Solomons, where it is sympatric with the larger species *P. mahaganus* (Ysabel, Choiseul, Bougainville), which falls in the CBL size range that *P. admiralitatum* exhibits on other islands (figure 1; electronic supplementary material, figure S7). This kind of pattern of intraspecific variation correlated with the presence and size of sympatric species is also observed in *P. neohibernicus*, one of the largest pteropodines. In this species, larger individuals of the subspecies *P. n. neohibernicus* are found where the species range overlaps with *P. conspicillatus*, another large species (mainland New Guinea, Bismarck Archipelago), but *P. neohibernicus hilli*, from the Admiralty Islands, where *P. conspicillatus* is not present, is significantly smaller (electronic supplementary material, figure S7).

## 4. Discussion

### (a) Phylogeny and biogeography

Here, we present an updated phylogenetic tree for pteropodines that is the most taxonomically complete group to date. Our results recovered previously proposed tribes, genera and *Pteropus* species groups [13,16,18]. The addition of more taxa to the analysis granted the modification of some species groups to include more species and the erection of new, single species groups (electronic supplementary material). Also worth mentioning are the conflicting relationships uncovered for the different specimens of *P. alecto* from Sulawesi and Australia (see electronic supplementary material for further discussion).

Our phylogenetic analysis confirms previous findings suggesting that sympatry of *Pteropus* species is the result of multiple colonization events rather than a product of sympatric speciation [16,18]. In a handful of cases, sister species pairs co-occur broadly in the same region, such as *P. neohibernicus* (New Guinea and the Bismarck Archipelago) and *P. conspicillatus* (Moluccas, New Guinea, and eastern Australia), which are sympatric in New Guinea, or *P. woodfordi* and *P. mahaganus*, which are endemic to the Solomon Islands. In the latter case, however, where they ‘co-occur’, *P. mahaganus* lives mainly on the larger islands while *P. woodfordi* lives mainly on adjacent small offshore islands. In all of these cases, each species occurs on many islands, implying that speciation could also have occurred in allopatry, as for other *Pteropus* species, with sympatry being attained through secondary contact (speciation followed by dispersal). These sister species pairs also showed marked size differences, and, according to our molecular dating analysis, diverged at least 1 million years ago.

### (b) Phylogenetic and geographical patterns of body size distribution

In most islands or groups of islands where pteropodines occur, the distribution of sizes among species is non-random and tends to be non-overlapping as first documented in the beginning of the 20th century by Andersen [31]. Our comprehensive dataset of CBL measurements allowed us to statistically evaluate this observation and confirm it. This is largely maintained even on species-rich islands where interspecific size differences appeared more finely subdivided. Our reconstruction of the history of pteropodine size evolution in a phylogenetic context showed several instances of size shifts, involving both size increases and decreases, as well as significant size disparity within clades, which suggests non-random evolution of size in *Pteropus* and more generally in frugivorous pteropodines.

To better understand the evolution of body size in the family Pteropodinae and evaluate hypotheses about drivers, we classified species into categories based on relative body size and sympatry status. ‘Small’ *Pteropus* species were scattered throughout the phylogeny, although they were represented most strongly in the *pelagicus* species group and in various monotypic species groups with long internal branches (i.e. *P. lombocensis*, *P. personatus*, *P. scapulatus*, *P. vetulus* and *P. temminckii*). Many (but not all) of these ‘small’ species are known or suspected to have diets including substantial percentages of floral parts in addition to fruits, which further reduces niche overlapping with larger, more frugivorous species [57–59]. Because the size category assignment was relative to the other species that occupy the island, the ‘medium’ category overlapped with the ranges of sizes of both ‘small’ and ‘large’ species across the phylogeny. The average CBL of ‘medium’ species was close to the estimated ancestral CBL for pteropodines (excluding Melonycterini) and the genus *Pteropus*. While ‘medium’ and ‘small’ species were evenly spread among the different clades of the phylogeny, the ‘large’ category included species from fewer *Pteropus* species groups overall. Islands where the ‘large’ species had a size in the highest range of the category also had relatively big ‘medium’ species (e.g. *P. melanopogon* and *P. chrysoproctus* as ‘large’ and ‘medium’, respectively, in the south Moluccas, and *P. neohibernicus* and *P. conspicillatus* as ‘large’ and ‘medium’, respectively, in New Guinea.

In one of the alternative schemes tested, we included in the ‘medium’ category isolated species that do not share their distribution with other pteropodines, because we reasoned that in the absence of selective pressure from competition, they might more likely conserve the ancestral size. Nevertheless, schemes where those species were considered to be in a category of their own were a better fit to the data. The ‘isolated’ species had an average CBL (57.2 mm ± 9.3) that was very similar to the estimated ancestral CBL, but with a wider variation than the one found among sympatric ‘medium’ species. The ‘isolated’ CBL range encompassed the entire range of the ‘small’ and ‘medium’ categories, and part of the ‘large’ category range (figure 2). Many of those isolated species inhabit small, isolated islands, which can favour drastic morphological evolution following a founder effect and differential ecological adaptation in mammals [60].

Some of the ‘large’ *Pteropus* species have widespread distributions in the sense that they are present in more than one archipelago (e.g. *P. tonganus* and *P. vampyrus*, which occur widely over various major regions), consistent with ideas of the hypothesis that large size may facilitate dispersal [30]. However, there are very large species that are endemic to more limited regions (e.g. *Acerodon jubatus* and *P. neohibernicus*). At the same time, one of the most widespread species, *P. hypomelanus*, usually falls in the ‘medium’ size category on islands. In any case, it is clear that dispersal ability is not solely dictated by body size in *Pteropus* and dispersal ability is also influenced by wing morphology and specific flight abilities [61]. Several of the widespread *Pteropus* species are phylogenetically close to species that occur in isolated islands of Micronesia and in the Indian Ocean, which is consistent with enhanced dispersal abilities in those clades relative to other *Pteropus* lineages (i.e. allowing them to initially colonize far-flung islands and archipelagos). The *griseus* group, in particular, encompasses most of the widespread species, including both ‘medium’ and ‘large’ sized ones. Some widespread species of this group, such as *P. griseus* and *P. admiralitatum,* have unresolved taxonomic issues and may actually represent species clusters (see comments about *P. alecto* in the electronic supplementary material). *Pteropus admiralitatum*, for instance, has populations in the Solomons, Bismarcks and Admiralty Islands with significant size and other morphological differences between them. More detailed studies are needed to determine the degree of genetic isolation and phylogenetic relationships of those populations.

### (c) Mechanisms of size evolution in *Pteropus*

The pattern of little or no overlap in body sizes among sympatric flying foxes could have different explanations, including chance, species sorting, character displacement and combinations of these different processes. The use of phylogenetic comparative methods, which allows comparing the fit of different models of phenotypic evolution to the data while taking into account the species tree, can give us clues about the main process(es) accounting for the observed pattern. The model with best fit to the evolution of size, as estimated by CBL, in *Pteropus* and pteropodines was the Hansen model, i.e. an OU model with different arrangements of optima along the branches of a phylogeny. Specifically, the best fitting model had four optima, to which species responded according to sympatry with other pteropodine species. It is possible that other, more complex schemes (with more optima), not assessed here, could have an even better fit, although our dataset may not be large enough to be reliably fit into more complex models. In any case, our results reject chance as the main mechanism to explain the observed pattern and strongly suggest that non-neutral processes were important drivers of evolutionary change in CBL.

Species sorting (also known as biased extinction) occurs when two species that are ecologically very similar come to exist in sympatry, e.g. colonizing the same island, and one of them disappears from the area of sympatry soon after the colonization event due to competition [62,63]. This process does not involve specific phenotypic changes in response to sympatry, but instead it assumes that the evolution of species phenotypes occurred independently and prior to the event of species coming into contact. For pteropodines, that would mean that size evolution does not occur after arrival on an island, and whatever change that does occur is independent of the presence or absence of other species. Therefore, less intra-clade (i.e. recent) phenotypic variation and more phylogenetic signal would be expected, a pattern that would most likely have a better fit to a Brownian motion model (instead of an OU model). This explanatory model is not a good fit to our dataset, in which intraspecific size differences suggest rapid size changes following colonization of an island in response to the sizes of sympatric species. Nevertheless, species sorting cannot be completely ruled out as a process operating in some situations for pteropodines; our body size data do show notable phylogenetic signal in some groups, with smaller species being more common in certain clades, for instance.

Character displacement is a coevolutionary process that involves natural selection directed towards trait values that will minimize niche overlapping between sympatric species [33,64]. The ‘species-for-species matching pattern’ (similar phenotype distributions in different islands and communities) and the overdispersion of trait means (even distribution of trait means) observed in some islands are patterns often attributed to a process of evolution by character displacement [33]. This process is compatible with the data fitting better to a multiple-optima OU model, since it would involve selection towards different, often opposing size optima, i.e. differential directional selection. Evidence that supports character displacement in pteropodines includes the distribution of species in different categories across the phylogeny, and our finding that changes in body size from ancestral states had different trends in the different categories of species (i.e. the smallest species in sympatric communities showed a trend in size reduction since speciation, the largest species tended to increase in size, while medium species tended to maintain their ancestral size). In some species, there were size changes that appear to have occurred along relatively short time scales, at the subspecies and island population levels in the last million years, probably in response to the different communities of species found in sympatry on different islands. There is no definitive evidence to date, however, to prove two criteria for character displacement: that the observed phenotypic differences are genetic and that similar-sized species compete for resources [33]. However, it is generally accepted that size is heritable, and that these bats use fruit resources in their islands, so character displacement is defendable as a hypothesis for *Pteropus* and pteropodines.

Another process that influences size evolution is character release, which happens when a species moves into a new area where lack of competition allows a trait to evolve under other selective pressures, resulting in novel ways of resource use. Character release may explain the wider variation in size that we observed among isolated species compared to those occurring sympatrically with other pteropodines. The slight tendency to size reduction in the ‘isolated’ category of species is probably related to the fact that most such species inhabit small, isolated islands with limited resources, a situation which may favour smaller body sizes [30]. Also, we cannot rule out phenotypic plasticity in determining pteropodine body size on certain islands [65]. A good test for the role of phenotypic plasticity in defining body sizes across different islands would involve testing the correlation between quantitative genetic differences with size differences between populations, which is beyond the scope of our study.

Unfortunately, a few extant species were missing from our model-fitting analyses due to a lack of DNA sequences, including *P. melanopogon, P. keyensis* and *P. aruensis* (which may well be conspecific), as well as the eastern Indian Ocean taxa *P. faunulus* and *P. melanotus*. Also missing from our tree are five out of six recently extinct species (the exception being *P. tokudae*, which was included). Although CBL data and the geographical distribution of those species were taken into consideration when classifying species into categories, their absence from the phylogeny reduced the power of some analysis. Another possible caveat for our analyses involves the possibility that overlooked extinct species, so far unknown to science, may have influenced the evolutionary patterns of pteropodine body size on certain islands. Similarly, currently cryptic taxonomic variation within nominal species, especially in widespread species, such as *P. admiralitatum*, might also affect the efficacy of our analysis as they may represent multiple distinctive species that we currently recognize as a single species. For many species, more in-depth population genetic analysis could provide valuable data both to resolve ongoing taxonomic problems and to study the evolutionary processes involved in size evolution and niche partitioning that we have uncovered in our study of pteropodines.

### (d) Ecological mechanisms facilitating the sympatric occurrence of pteropodines

Across the diversity of *Pteropus* and its sister genus *Acerodon*, we found very few examples of species of the same body size class co-occurring on the same islands, a firm demonstration of the importance of body size distinction as a principal mechanism facilitating co-occurrence in flying fox species. Two exceptions noted in our analysis involve *P. hypomelanus* and closer examination of local geography provides important ecological context regarding these apparent co-occurrences. In both cases, *P. hypomelanus* is a species that is usually found only on smaller islets and coastal margins within the archipelagos in question. This small-island specialization, which is general within the ‘*hypomelanus* group’ of flying foxes, appears to facilitate archipelago-level sympatry with similar sized species, while at the same time reducing direct co-occurrence (syntopy) with species like *A. celebensis*, which is more affiliated with mainland Sulawesi and large satellite islands (rather than small islands), or like *P. caniceps*, which is mostly recorded from larger north Moluccan islands, rather than smaller offshore islands. This local ecological segregation of similar size species to islands of different sizes within archipelagos is the best documented means of facilitating ecological co-occurrence in flying foxes apart from body size distinctions.

Unlike the pattern in *Pteropus* and *Acerodon*, species in genera of the tribe Pteralopini seem to be well adapted to co-existing with similar-sized species on islands, both with other Pteralopini species and with *Pteropus* species. But remarkably, previous studies have suggested that Pteralopini species seem to have altitudinally stratified distributions and specific habitat preferences: for example, in the Solomon Islands, while *Pteralopex atrata*, *P. taki*, and *P. flanneryi* seem to occur only at low elevations, *P. anceps* and *P. pulchra* inhabit montane forests [66]. Thus, differential elevational preferences probably facilitate the co-occurrence of *P. pulchra* with *P. atrata*, and with additional species of *Pteropus*, on Guadalcanal, and the co-occurrence of *P. anceps* with *P. flanneryi* and with additional species of *Pteropus*, on Bougainville and Choiseul. While they are probably usually elevationally stratified, nevertheless *Pteralopex flanneryi* and *P. anceps* have been documented sympatrically at low elevations on the large island of Bougainville, where they appear to show only a small difference in size but probably differ substantively in feeding ecology, as indicated by the relatively much larger incisors but smaller molars of *P. anceps* as compared with those of *P. flanneryi* [66]. Similarly, *M. acrodonta* is only found in montane mossy forests in Taveuni Island in Fiji and has not been found at lower elevations, together with *Pteropus samoensis* and *P. tonganus*. These indications provide an important clue that within Pteralopini, elevational and major ecomorphological distinctions may be more important in facilitating co-occurrence with other pteropodines (including with *Pteropus*). We suspect that similar factors may be important too in the biology of *Desmalopex* in the Philippines. Unfortunately, all Pteralopini species are relatively rare and little-studied, such that major taxonomic boundaries in the group have only recently been illuminated [66–68]; little is known about their ecology, and only a few specimens were available for measurement. Other factors and processes may also have contributed to the observed patterns of size distributions in pteropodines. For instance, because body size and island/archipelago/landmass size are (albeit weakly) correlated (see [30]), it is possible that the area of the island imposes size limits on the entire community of species that inhabit it.

## Data Availability

The data used is provided in the supplementary tables. Supplementary material is available online [69].

## References

[1] Giannini NP. 2019 Pteropodidae. In Handbook of the mammals of the world (eds DE Wilson, R Mittermeier), pp. 16–61. Barcelona, Spain: Lynx Edicions.

[2] Simmons NB, Cirranello AL. 2024 Bat species of the world: a taxonomic and geographic database. See https://batnames.org/.

[3] Freeman PW. 1995 Nectarivorous feeding mechanisms in bats. Biol. J. Linn. Soc. **56**, 439–463. (10.1111/j.1095-8312.1995.tb01104.x)

[4] Kirsch JA, Lapointe FJ. 1997 You aren’t (always) what you eat: evolution of nectar-feeding among Old World fruitbats (Megachiroptera: Pteropodidae). Mol. Evol. Adapt. Radiat. 313–330.

[5] Banack SA. 1998 Diet selection and resource use by flying foxes (genus Pteropus). Ecology **79**, 8. (10.1890/0012-9658(1998)079[1949:dsarub]2.0.co;2)

[6] Hodgkison R, Balding ST, Zubaid A, Kunz TH. 2003 Fruit bats (Chiroptera: Pteropodidae) as seed dispersers and pollinators in a lowland Malaysian rain forest. Biotropica **35**, 491–502. (10.1111/j.1744-7429.2003.tb00606.x)

[7] Raghuram H, Singaravelan N, Nathan PT, Rajan KE, Marimuthu G. 2011 Foraging ecology of pteropodid bats: pollination and seed dispersal. In Bats: biology, behavior and conservation (eds JL Zupan, SL Mlakar), pp. 177–188. Hauppauge, NY: Nova Science Publishers.

[8] Fujita MS, Tuttle MD. 1991 Flying foxes (Chiroptera: Pteropodidae): threatened animals of key ecological and economic importance. Conserv. Biol. **5**, 455–463.

[9] Helgen KM, Helgen LE, Wilson DE. 2009 Pacific flying foxes (Mammalia: Chiroptera): two new species of Pteropus from Samoa, probably extinct. Am. Mus. Novit. **3646**, 1–37. (10.1206/614.1)

[10] Vincenot CE, Florens FBV, Kingston T. 2017 Can we protect island flying foxes? Science **355**, 1368–1370. (10.1126/science.aam7582) 28360279

[11] Kingston T, Florens FBV, Vincenot CE. 2023 Large Old World fruit bats on the brink of extinction: causes and consequences. Annu. Rev. Ecol. Evol. Syst. **54**, 237–257. (10.1146/annurev-ecolsys-110321-055122)

[12] Upham NS, Esselstyn JA, Jetz W. 2019 Inferring the mammal tree: species-level sets of phylogenies for questions in ecology, evolution, and conservation. PLoS Biol. **17**, e3000494. (10.1371/journal.pbio.3000494) 31800571 PMC6892540

[13] Almeida FC, Amador LI, Giannini NP. 2021 Explosive radiation at the origin of Old World fruit bats (Chiroptera, Pteropodidae). Org. Divers. Evol. **21**, 231–243. (10.1007/s13127-021-00480-5)

[14] Almeida FC, Giannini NP, DeSalle R, Simmons NB. 2011 Evolutionary relationships of the Old World fruit bats (Chiroptera, Pteropodidae): another star phylogeny? BMC Evol. Biol. **11**, 281. (10.1186/1471-2148-11-281) 21961908 PMC3199269

[15] Almeida FC, Simmons NB, Giannini NP. 2020 A species-level phylogeny of Old World fruit bats with a new higher-level classification of the family Pteropodidae. Am. Mus. Novit. **2020**, 1. (10.1206/3950.1)

[16] Tsang SM, Wiantoro S, Veluz MJ, Sugita N, Nguyen Y, Simmons NB, Lohman DJ. 2020 Dispersal out of Wallacea spurs diversification of Pteropus flying foxes, the world’s largest bats (Mammalia: Chiroptera). J. Biogeogr. **47**, 527–537. (10.1111/jbi.13750) 33041434 PMC7546435

[17] Hall R. 2002 Cenozoic geological and plate tectonic evolution of SE Asia and the SW Pacific: computer-based reconstructions, model and animations. J. Asian Earth Sci. **20**, 353–431. (10.1016/s1367-9120(01)00069-4)

[18] Almeida FC, Giannini NP, Simmons NB, Helgen KM. 2014 Each flying fox on its own branch: a phylogenetic tree for Pteropus and related genera based on DNA sequences. Mol. Phylogenetics Evol. **77**, 83–95. (10.1016/j.ympev.2014.03.009) 24662680

[19] Nowak RM. 1994 Walker’s bats of the world. Baltimore, MD: John Hopkins University Press.

[20] Hintsche S. 2019 *Pteropus molossinus*. In Handbook of the mammals of the world (eds DE Wilson, RA Mittermeier). Barcelona, Spain: Lynx Editions.

[21] Jones G. 1999 Scaling of echolocation call parameters in bats. J. Exp. Biol. **202**, 3359–3367. (10.1242/jeb.202.23.3359) 10562518

[22] Simmons NB, Conway T. 2003 Evolution of ecological diversity in bats. In Bat ecology (eds TH Kunz, MB Fenton). Chicago, IL: University of Chicago Press.

[23] Moyers-Arévalo RL, Amador LI, Almeida FC, Giannini NP. 2018 Evolution of body mass in bats: insights from a large supermatrix phylogeny. J. Mamm. Evol. (10.1007/s10914-018-9447-8)

[24] Hand SJ, Maugoust J, Beck RMD, Orliac MJ. 2023 A 50-million-year-old, three-dimensionally preserved bat skull supports an early origin for modern echolocation. Curr. Biol. **33**, 4624–4640.(10.1016/j.cub.2023.09.043) 37858341

[25] Mayr E. 1963 Animal species and evolution. Cambridge, MA: Harvard University Press.

[26] Woolfit M, Bromham L. 2005 Population size and molecular evolution on islands. Proc. Biol. Sci. **272**, 2277–2282. (10.1098/rspb.2005.3217) 16191640 PMC1560185

[27] Losos JB, Ricklefs RE. 2009 Adaptation and diversification on islands. Nature **457**, 830–836. (10.1038/nature07893) 19212401

[28] Foster JB. 1964 Evolution of mammals on islands. Nature **202**, 234–235.

[29] Van Valen L. 1973 Body size and numbers of plants and animals. Evolution **27**, 27–35. (10.1111/j.1558-5646.1973.tb05914.x) 28563673

[30] Lomolino MV. 2005 Body size evolution in insular vertebrates: generality of the island rule. J. Biogeogr. **32**, 1683–1699. (10.1111/j.1365-2699.2005.01314.x)

[31] Andersen K. 1912 Catalogue of the chiroptera in the collection of the British Museum, vol. 1, 2nd edn. London, UK: Megachiroptera. Trustees British Museum.

[32] Dayan T, Simberloff D. 1998 Size patterns among competitors: ecological character displacement and character release in mammals, with special reference to island populations. Mammal Rev. **28**, 99–124. (10.1046/j.1365-2907.1998.00029.x)

[33] Schluter D 2000 Ecological character displacement in adaptive radiation. Am. Nat. **156**, S4. (10.2307/3079223)

[34] Wilson DE, Mittermeier RA. 2019 Handbook of the mammals of the world. In Bats, p. 1008, vol. **9**. Barcelona, Spain: Lynx Editions.

[35] Marsh CJ *et al*. 2022 Expert range maps of global mammal distributions harmonised to three taxonomic authorities. J. Biogeogr. **49**, 979–992. (10.1111/jbi.14330) 35506011 PMC9060555

[36] Welbergen JA. 2010 Growth, bimaturation, and sexual size dimorphism in wild gray-headed flying foxes (Pteropus poliocephalus). J. Mammal. **91**, 38–47. (10.1644/09-MAMM-A-157R.1)

[37] Andrianiaina A, Andry S, Gentles A, Guth S, Héraud JM, Ranaivoson HC, Ravelomanantsoa NAF, Treuer T, Brook CE. 2022 Reproduction, seasonal morphology, and juvenile growth in three Malagasy fruit bats. J. Mammal. **103**, 1397–1408. (10.1093/jmammal/gyac072) 36686611 PMC9841406

[38] Jones KE *et al*. 2009 PanTHERIA: a species‐level database of life history, ecology, and geography of extant and recently extinct mammals. Ecology **90**, 2648–2648. (10.1890/08-1494.1)

[39] R.Core Team. 2021 R: a language and environment for statistical computing. Vienna, Austria: R Foundation for Statistical Computing. See https://www.R-project.org/.

[40] Katoh K, Standley DM. 2013 MAFFT multiple sequence alignment software version 7: improvements in performance and usability. Mol. Biol. Evol. **30**, 772–780. (10.1093/molbev/mst010) 23329690 PMC3603318

[41] Maddison W, Maddison D. 2007 Mesquite 2. A modular system for evolutionary analysis. See http://www.mesquiteproject.org.

[42] Nguyen LT, Schmidt HA, von Haeseler A, Minh BQ. 2015 IQ-TREE: a fast and effective stochastic algorithm for estimating maximum-likelihood phylogenies. Mol. Biol. Evol. **32**, 268–274. (10.1093/molbev/msu300) 25371430 PMC4271533

[43] Drummond AJ, Rambaut A. 2007 BEAST: Bayesian evolutionary analysis by sampling trees. BMC Evol. Biol. **7**, 214. (10.1186/1471-2148-7-214) 17996036 PMC2247476

[44] Amador LI, Moyers Arévalo RL, Almeida FC, Catalano SA, Giannini NP. 2018 Bat systematics in the light of unconstrained analyses of a comprehensive molecular supermatrix. J. Mamm. Evol. **25**, 37–70. (10.1007/s10914-016-9363-8)

[45] Pagel M. 1999 Inferring the historical patterns of biological evolution. Nature **401**, 877–884. (10.1038/44766) 10553904

[46] Blomberg SP, Garland T, Ives AR. 2003 Testing for phylogenetic signal in comparative data: behavioral traits are more labile. Evolution **57**, 717–745. (10.1111/j.0014-3820.2003.tb00285.x) 12778543

[47] Revell LJ. 2012 phytools: an R package for phylogenetic comparative biology (and other things). Methods Ecol. Evol. **3**, 217–223. (10.1111/j.2041-210x.2011.00169.x)

[48] Paradis E, Schliep K. 2019 ape 5.0: an environment for modern phylogenetics and evolutionary analyses in R. Bioinformatics **35**, 526–528. (10.1093/bioinformatics/bty633) 30016406

[49] Felsenstein J. 1985 Phylogenies and the comparative method. Am. Nat. **125**, 1–15. (10.1086/284325)

[50] Harmon LJ *et al*. 2010 Early bursts of body size and shape evolution are rare in comparative data. Evolution **64**, 2385–2396. (10.1111/j.1558-5646.2010.01025.x) 20455932

[51] Hansen TF. 1997 Stabilizing selection and the comparative analysis of adaptation. Evolution **51**, 1341–1351. (10.1111/j.1558-5646.1997.tb01457.x) 28568616

[52] Pennell MW, Eastman JM, Slater GJ, Brown JW, Uyeda JC, FitzJohn RG, Alfaro ME, Harmon LJ. 2014 geiger v2.0: an expanded suite of methods for fitting macroevolutionary models to phylogenetic trees. Bioinformatics **30**, 2216–2218. (10.1093/bioinformatics/btu181) 24728855

[53] Akaike H. 1974 A new look at the statistical model identification. IEEE Trans. Autom. Control **19**, 716–723.

[54] Butler MA 2004 Phylogenetic comparative analysis: a modeling approach for adaptive evolution. Am. Nat. **164**, 683. (10.2307/3473229) 29641928

[55] Beaulieu JM, O’Meara B. 2022 OUwie: analysis of evolutionary rates in an OU framework. R package version 2.10.

[56] Revell LJ, Harmon LJ. 2022 Phylogenetic comparative methods in R. Princeton, NJ: Princeton University Press.

[57] Flannery TF. 1995 Mammals of new guinea. Sydney, Australia: Reed.

[58] Bonaccorso FJ. 1998 Bats of papua new guinea. Washington, DC: Conservation International.

[59] Buden D, Helgen KM, Wiles G. 2013 Taxonomy, distribution, and natural history of flying foxes (Chiroptera, Pteropodidae) in the Mortlock Islands and Chuuk State, Caroline Islands. ZooKeys **345**, 97–135. (10.3897/zookeys.345.5840) PMC3817444 24194666

[60] Thorpe RS, Surget-Groba Y, Johansson H. 2010 Genetic tests for ecological and allopatric speciation in anoles on an island archipelago. PLoS Genet. **6**, e1000929. (10.1371/journal.pgen.1000929) 20442860 PMC2861690

[61] Olival KJ. 2008 Population genetic structure and phylogeography of southeast Asian flying foxes: implications for conservation and disease ecology. Thesis, Columbia University, New York, NY.

[62] Strong DR, Szyska LA, Simberloff DS. 1979 Tests of community-wide character displacement against null hypotheses. Evolution **33**, 897–913. (10.1111/j.1558-5646.1979.tb04743.x) 28568434

[63] Roughgarden J. 1983 Competition and theory in community ecology. Am. Nat. **122**, 583–601. (10.1086/284160)

[64] Brown WL, Wilson EO. 1956 Character displacement. Syst. Zool. **5**, 49–64.

[65] Cooper DM, Yamaguchi N, Macdonald DW, Nanova OG, Yudin VG, Dugmore AJ, Kitchener AC. 2022 Phenotypic plasticity determines differences between the skulls of tigers from mainland Asia. R. Soc. Open Sci. **9**, 220697. (10.1098/rsos.220697) 36465684 PMC9709513

[66] Helgen KM. 2005 Systematics of the Pacific monkey‐faced bats (Chiroptera: Pteropodidae), with a new species of Pteralopex and a new Fijian genus. System. Biodivers. **3**, 433–453. (10.1017/S1477200005001702)

[67] Giannini NP, Almeida FC, Simmons NB, Helgen KM. 2008 The systematic position of Pteropus leucopterus and its bearing on the monophyly and relationships of Pteropus (Chiroptera: Pteropodidae). Acta Chiropterologica **10**, 11–20. (10.3161/150811008x331054)

[68] Esselstyn JA, Garcia HJD, Saulog MG, Heaney LR. 2008 A new species of Desmalopex (Pteropodidae) from the Philippines, with a phylogenetic analysis of the Pteropodini. J. Mammal. **89**, 815–825. (10.1644/07-mamm-a-285.1)

[69] Almeida F, Helgen KM, Simmons N, Giannini NP. 2025 Supplementary material from: Evolution and ecology of body size in the world’s largest bat. Figshare. (10.6084/m9.figshare.c.7908465)40664244

